# Structure Characterization and Products Control of Technical Chlorinated Paraffins by Direct Injection Mass Spectrometry With Data Deconvolution and ^1^H NMR With Chemometrics Tools

**DOI:** 10.1155/jamc/1180345

**Published:** 2025-04-19

**Authors:** Zhouman Huang, Yan Liu, Haipeng Jiang

**Affiliations:** ^1^College of Innovation and Entrepreneurship, Wuchang University of Technology, Wuhan, Hubei 430223, China; ^2^Medical and Nursing School, Wuhan Railway Vocational College of Technology, Wuhan, Hubei 430205, China; ^3^School of Chemical Engineering and Pharmacy, Pharmaceutical Research Institute, Wuhan Institute of Technology, Wuhan, Hubei 430205, China

**Keywords:** ^1^H NMR, chlorinated paraffins, direct injection mass spectrometry, quality control, structure characterization

## Abstract

The diverse industrial use of chlorinated paraffins (CPs) have led to their environmental dispersion, and special attention has been paid to their ecotoxicology. Among them, short-chain chlorinated paraffins (SCCPs) have been listed as potential persistent organic pollutants (POPs). However, currently, technical CPs produced by manufacturers are usually labeled by their chlorination degree, but such structural label is not enough to reflect CPs' environmental fate and toxicity. Ecotoxicology research suggested that the chain length, chlorination degree and the chlorine distribution pattern are all factors that can determine CPs' environmental fate and toxicity. Herein, we present a cost-effective method for the structure characterization of technical CPs. By using direct injection mass spectrometry with data deconvolution, chain length distribution and homologous distribution in technical CPs mixture can be delineated. By using ^1^H NMR with chemometrics tools, the chlorine distribution pattern can be elaborated. Combining the abovementioned two analytical strategies, structural information at different levels that related to CPs' environmental fate and toxicities were revealed. This method is expected to be easily applied in both industry and academia, aiming for quality control of technical CPs, by permitting only nontoxic or noncarcinogenic CPs into industrial use.

## 1. Introduction

Chlorinated paraffins (CPs), are complex mixtures of polychlorinated alkanes, produced by free radical chain reactions involving chlorine and n-alkanes. The global production capacity of CPs is above 3 million tons, and China account for 65% of this global production capacity [1, 2]. As versatile chemical products, CPs have the characteristic of low volatility, excellent flame retardancy, and electrical insulation, and they can be used as flame retardants and plasticizers in different material, such as plastics, rubber, and fibers [3]. Such diverse applications of CPs have given rise to widespread environmental dispersion. The major releases are thought to originate from its industrial use in an open environment. Besides, disposal and burning of waste containing CPs are additional potential pathways for entry into environmental migration [1, 4, 5].

Ecotoxicological studies have conveniently classified CPs on the basis of their chain lengths, as short-chain chlorinated paraffins (SCCPs), medium-chain chlorinated paraffins (MCCPs), and long-chain chlorinated paraffins (LCCPs). Generally speaking, SCCPs have a linear chain of C_10_–C_13_, MCCPs have a linear chain of C_14_–C_17_, and LCCPs have a linear chain length ≥ 18. CPs are highly bioaccumulative and not easily degradable. The industrial use of CPs can easily lead to their transfer and amplification in the environment and food chain. CPs have been detected in a wide range of environmental media, such as air, water, sediments, soils, and biota [6]. Toxicological research disclosed that the short-chain, heavily chlorinated paraffin have a greater potential for chronic toxicity and carcinogenicity than the longer-chain, lightly chlorinated paraffin [6, 7]. It can be concluded that the physical properties and toxicity of CPs are closely related to its molecular structure and follow a certain structure-activity relationship. The chain length, the percentage of chlorination, and the chlorine distribution pattern are all the factors which determine CPs' environmental fate and toxicity.

In the industrial production, the intended chlorination degree is controlled by the raw material feeding ratio. Technical CPs are usually labeled as chlorination degree, such as CP-42, CP-52, and CP-70, meaning the chlorination degree are 42%, 52%, and 70%, respectively. Among these CPs products, CP-52 accounts for nearly 80% of the total commercial production [2, 3]. This current technical CPs' labeling method only focuses on its gross chlorine percentage, it differs from regulatory classifications, which according to CPs' chain lengths, namely, SCCPs, MCCPs, and LCCPs [8, 9]. As mentioned above that the chain length, the percentage of chlorination and the chlorine distribution pattern are all the factors which determe CPs' environmental fate and toxicity. It can be inferred that nomenclature using chlorine percentage and chain length can not reflecting CPs toxicity, and further structure characterization are needed.

Due to the known toxicity of SCCPs, they have been listed as potential persistent organic pollutants (POPs) since May 2017. However, at that moment, many of the various parties that signed the Stockholm Convention on the “dirty dozen” have not yet approved the ban of novel POPs including SCCPs [3]. Recently, China signed the act of the “Stockholm Convention on Persistent Organic Pollutants Amendment to the list of three categories of persistent organic pollutants including short-chain chlorinated paraffins,” on December 30, 2022 [10]. In the case of MCCPs and LCCPs, relevant data on their environmental fate and potential toxicity are still insufficient to facilitate international regulations. So, MCCPs and LCCPs are currently not regulated [11]. However, in technical MCCPs and LCCPs, a limit for the presence of SCCPs was set at 1.0% by weight [12].

As mentioned above, CPs' contamination is thought to be mainly from industrial use, disposal, and burning. In order to control pollution from the source and to avoid toxic CPs entering the downstream process of industrial use, it is important to quality control of technical CPs before its market access, permitting only nontoxic or noncarcinogenic CPs to enter industrial use. So, an analytical method for structure characterization of technical chlorinated paraffins, aiming to delineate the chain length, chlorination degree, and the chlorine distribution pattern, is meaningful for product control of technical CPs. However, structure characterization of CPs is a challenging topic in environmental analytics. Because CPs are produced by free radical chain reactions involving chlorine and n-alkanes, such free radical reactions show low positional selectivity and produce innumerable congeners and isomers. A typical CP is an extremely complex mixture with the chemical structure of polychlorinated n-alkanes (i.e., C_n_H_2n+2−m_Cl_m_), with different chain lengths and chlorination substitution patterns [5]. To date, there is a scarcity of works concerning attempts for the chromatographic separation of CPs and their semiquantitation of congener in such highly complex mixtures [13–16]. Therefore, the fully structure analysis of each congener is infeasible [17, 18].

The goal of this study is to explore the opportunities of building a cost-effective structure characterization method for quality control of CPs, aiming to delineate the chain length, chlorination degree, and the chlorine distribution pattern. In this method, direct injection low resolution mass spectrometry (LR-MS) with data deconvolution was used to delineate chain length distribution and homologous distribution in technical CP-52 samples; in the method, chloride-enhanced atmospheric pressure chemical ionization (APCI)-MS ionization was chosen, considering for its uniformity of ionization and its applicablity to the low vapor pressure of the samples [19] and such ionization method is suitable for direct injection. Meanwhile, ^1^H NMR with chemometric tools was used to elaborate the chlorine distribution pattern in the carbon chain. By combining the abovementioned two strategies, structural information at different levels related to CPs' environmental fate and toxicity were revealed. This method is expected to be easily applied in both industry and academia, aiming for quality control of technical CPs, to permit only CPs of nontoxic or noncarcinogenic to enter industrial use.

## 2. Materials and Methods

### 2.1. Reagents and Chemicals

Seven technical CP-52 samples were obtained from five different manufacturers, marked as S1, S2, S3, YT, Z-52, N26, and L647. Among them, S1, S2, and S3 were different batches from a same manufacturer, and other products were individually from different manufacturers. The abovementioned samples were all labeled as CP-52 according to their chlorine percentage (52% Cl, w/w), while chain length information and structure information were not declared.

### 2.2. Direct Injection LR-APCI-MS

100 μL each CP sample was dissolved in 2 mL CH_2_Cl_2_ (HPLC‐grade), then the CP solution was drawn into a syringe and directly injected into LR-APCI-MS analysis with the speed of 20 μL/second using a peristaltic pump.

LR-APCI-MS analysis was performed on a Thermofisher LTQ XL instrument in negative ion mode, with full scan range was set to m/z 50–1000. The key operation parameter as following: the capillary temperature was set at 300°C, APCI vaporizer temperature was set at 350°C, and the discharge voltage was set at 4.0 kV. Nitrogen was used as nebulizer gas, auxiliary gas, and sweep gas, and their flow rates were set (in arbitrary units/minute) at 30, 5, and 5, respectively.

### 2.3. Data Processing for LR-APCI-MS

Raw MS spectral data were exported as ASCII format (including extract mass and intensity of each peak) and then the data were imported into Origin 8.0 software (Origin Lab) with suitable format. Peak fitting was carried out by the “Peak Fit Wizard” module, with profile corresponding to each congener of CPs was fitted by Gaussian function. The relative content of each congener was calculated by the height of curve.

### 2.4. ^1^H NMR

Twenty μL each CP samples were dissolved in 0.5 mL CDCl_3_ (Sigma-Aldrich Co., contained 0.05 wt% TMS as a chemical shift reference) and then each prepared solution was transferred to standard 5 mm NMR tube. Proton nuclear magnetic resonance spectra were measured at 400.13 MHz using a Bruker AVANCE NEO 400 MHz instrument (Bruker, Karlsruhe, Germany), equipped with a 5 mm Smart Probe. ^1^H NMR experiments were conducted at 303 K, and spectral width was set of 20.0 ppm with O_1_P at 4.7 ppm, each with scan times of 32 s. Line broadening of 0.3 Hz was applied before Fourier transformation.

^1^H NMR spectra of each sample was carefully phased and baseline corrected. To reduce the quadrature, the spectra had the following sections cut:1.52–1.60 ppm, attributed to water; 1.17–1.40 ppm and 0.97–1.01 attributed to residual alkanes feedstock, and then the spectra range (0.55–6.40 ppm) was used to build multivariate models. Each matrix was preprocessed by bucketing with 0.03 ppm width; to normalize the intensities indifferent samples, buckets were scaled to the total intensity of 100. The excel data were then submitted to principal component analysis (PCA) unit of SIMCA-P 14.0 (Umetrics, Umea, Sweden). To investigate groupings and classification of the CP-52 samples from different manufacturers, the unsupervised analysis method of PCA was established. The unsupervised analysis method of artial least-squares-discriminant analysis (PLS-DA) was employed to search for potential different resonances. Finally, resonances with variable importance in projection (VIP) values sorted with value were deemed to be significant and important in distinguishing CP-52 samples from different sources.

## 3. Results and Discussion

### 3.1. Structure Characterization of Technical CPs by LR-APCI-MS

Technical CPs is complex mixtures, which is composed of a series of congeners, the formula of each congener can be denoted as C_n_H_2n+2−m_Cl_m_. As mentioned above, it has some difficlut to reach absolute identification and semiquantitation of each congeners, so in the present mehod, the combination the two approaches (one as direct-injection mass spectrometry and another NMR) is designed to delineate the structural characteristics of CP related to its ecological toxicity.

It has been showed that the sensitivity of the chlorine enhanced APCI source method was less dependent on the chlorination degree than other ionization methods, such as EI-MS, ECNI-MS and ESI-MS. Furthermore, [M + Cl]^−^ is the dominated pseudomolecular ions in the chlorine enhanced APCI ionization, which is beneficial for deconvolution [20, 21]. Besides, such ionization is applicable to the low vapor pressure of CP-52 samples [19] and suitable for direct injection. So, in the present strategy, chloride-enhanced APCI was chosen as ionization modes in the determination of CPs by MS.

Under the chloride-enhanced APCI mode, a series of isotope peak cluster of [M + Cl]^−^, that is, [C_n_H_2n+2−m_Cl_m+1_]^−^ with different combinations of n and m will be listed in its mass spectrum, isobaric interferences from isomers with the same m/z in the CPs mixture would lead to the overlap in the spectrum. There are some efforts in the deconvolution of isobaric interferences in overlapping pectrum [22, 23]. However, the reported methods relied on complicated deconvolution algorithm or expensive instruments, thus made it difficult to transfer to industry. Herein, we use LR-APCI-MS combining with data deconvolution to fulfill this goal.

**Figure 1:**
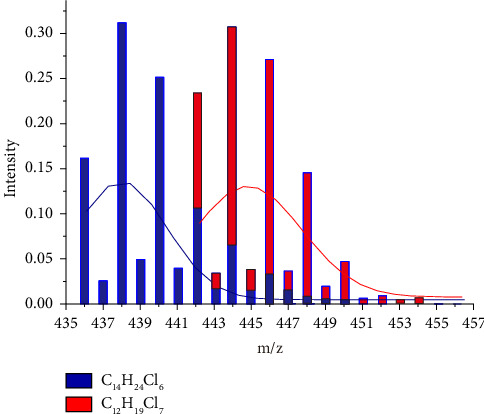
Simulated deconvolution result of C_14_H_24_Cl_6_ and C_12_H_19_C_l7_.

By simulating all possible saturated congeners in the range of C_12_–C_19_ using IsoPro 3.0, it was found that each congener has similar isotope peak cluster profile, with its peak maximum arrayed with the mass difference of 4 Da (m/z) or 6 Da (m/z) (see Supporting Information S1), which is attributed to the congener of C_n_H_2n+2−m_Cl_m+1_ and C_n+2_ H_2n-3−m_Cl_m+2_. In Figure 1, we use C_14_H_24_Cl_6_ (its isotope peak cluster denoted as blue) and C_12_H_19_Cl_7_ (its isotope peak cluster denoted as red) in 1:1 mixture as an example, and the peak distribution of [M + Cl]^−^ deduced from IsoPro 3.0 were introduced into Origin 8.0 software. Although some mass interferences from isobaric interferences were overlapped in its LR-MS mode, two Gaussian curves can be resolved with their maximum isotope peak or profile center at m/z 438 and m/z 444, with peak height ratio at 1:1 were clearly resolvled, by data deconvolution using the “Peak Fit Wizard” module. In this way, the deconvolution of overlapping regions can be achieved.

**Figure 2:**
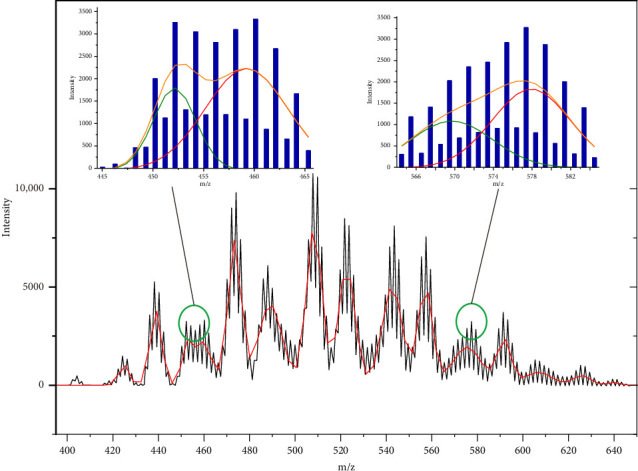
Deconvolution and fitting of LR-APCI-MS data of technical CP sample (Z-52 as example).

Then, the experimental mass spectra of real CP samples (Supporting Information Figure S1) were exported as ASCII format, and the data were then subjected to fitting using Origin Lab software. Z-52 was used as an example. Sixteen peaks were found using data deconvolution. Even overlapping regions at the range of m/z 445–465 and m/z 565–585 can also be deconvoluted and fitted, and the congeners with maximum isotope peak or profile center of mass difference at 4 Da (m/z) or 6 Da (m/z) were automatically found (see Figure 2). Then, the formula of each congener can be deduced from its m/z of profile center (referring to supporting information Table S2), and the content of each congener can be estimated by the peak height of Gaussian curve. In this way, carbon chain length distribution and homologous distribution of C_n_Cl_m_ in CP mixture can be reconstructed. Such data are meaningful for controlling and monitoring products quality of CPs, by referring to current regulatory classifications.

**Figure 3:**
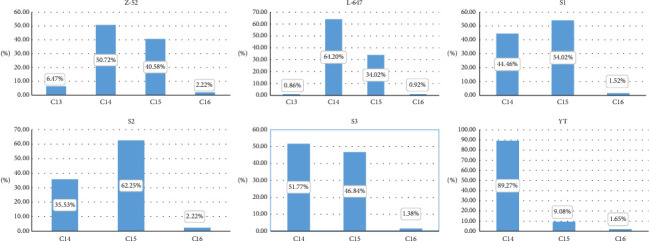
Carbon chain distribution of six technical CP samples.

As we know, CPs are produced through the chlorination of n-alkane raw materials, which are obtained from purified fractions obtained via large-scale industrial distillation. Because this purification process cannot isolate n-alkanes to single-chain length or remove undesired impurities sufficiently, the feedstocks consist of a mixture of homologs and thus the final mixture contains the diverse industrial CP products vary in the pattern of alkanes.  Figure 3 shows the carbon chain distribution of Z-52 and other technical CP-52 investigated by this research. It is indicated that that SCCPs coexisted in some MCCPs and LCCPs products, such result is consistent with previous research [24].

Apart from the carbon chain length distribution, relative amounts of each C_n_Cl_m_ can be also deduced from the fitting data. It has been found to follow normal distribution, according to their carbon chain length (referring to Supporting Information Table S2), and such result was also consistent with previous literature [12].

It is mentioned here that from the deconvolution result of N-26 (see Supporting Information Table S2), it may be containing 23.19% of C_12_ and 15.40% of C_13_ homologs. Besides, the carbon chain length ranges from C_12_–C_19_. These may originate from low-quality petroleum distillation fractions of alkane feedstocks for production. Based on our experience, the more complex the raw materials used as feedstocks, the more crowded the mass spectrum peak. Due to heavily overlapping mass spectra of N26, the accuracy of carbon chain distribution cannot be accurately obtained. It should be recognized that the method has its limitations on the resolving of CPs with complex carbon chains.

### 3.2. Structure Characterization of Technical CPs by NMR

In the above, LR-APCI-MS was employed as structure characterization tool for carbon chain length distribution and homologous distribution of C_n_Cl_m_ in technical CPs. However, further details such as structural elements about chlorination patterns on the carbon chain (including chlorine patterns of isomers and substitution position of chlorine in carbon chain) cannot be extracted from mass spectrometry. Such structure information is important for CPs' environmental toxicities [6]. The goal of this section was to explore the opportunities of NMR in the characterization and discrimination of structural elements of technical CP-52 mixtures.

**Figure 4:**
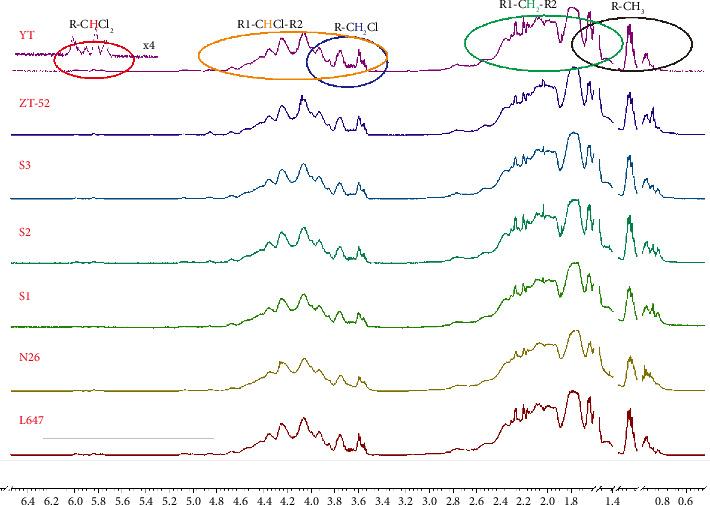
The ^1^H NMR spectra of the seven technical CP-52 samples.

Figure 4 shows the ^1^H NMR spectra of the seven technical CP-52 samples, which were plotted in the range of 0.5–6.5 ppm. By referring to the previous work about resonances assignment of CPs and my experience, the resonances are grouped in five major spectral ranges corresponding to R-CHCl_2_ (5.6–6.1 ppm, R, R_1_, or R_2_ denoted as carbon chain substituent containing 0–3 chlorine atoms, the same meaning in the following), R_1_-CHCl-R_2_ (3.4–5.3 ppm), R-CH_2_Cl (3.6–4.0 ppm), R_1_-CH_2_-R_2_ (1.3–2.8 ppm), and R-CH_3_(0.7–2.2 ppm) [18, 24, 25]. It is plausible that heavily chlorinated structural elements can be reflected by resonances signal in low field, such as resonance at 5.6–6.1 ppm can partially reflect heavily chlorinated structural elements; indeed, the structural elements with CCl_3_ at the terminal group may be the most heavily chlorinated ones, which can occur in the rest of spectral ranges except for the R-CHCl_2_ group. The major resonances were assigned in one of ^1^H NMR spectra of Figure 4.

By investigating the ^1^H NMR spectra of all seven technical CP-52 samples, it is found that the ^1^H NMR spectra are similar, especially the products from the same manufacturer. It is challenging for detecting such minor variability among ^1^H NMR spectra by visual inspection. In such cases, multivariate statistical analysis is the preferred method of constructing the model, which reflects all structural differences hidden in spectral data of CP-52 mixture from different spectra. So, PCA was tentatively employed to evaluate the product profile of CP-52.

**Figure 5:**
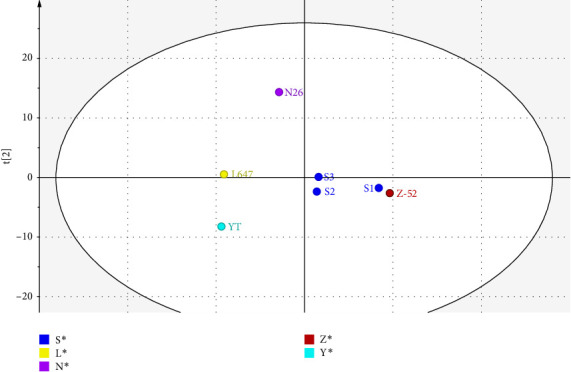
The PCA score plot of CP-52 samples from different manufacturers.

The PCA score plot of CP-52 samples from different manufacturers is shown in Figure 5. It shows clear clusters or differences among samples from different manufacturers. The first two components encompass 71.9% of the total variance within the dataset. In each cluster, batches from the same manufacturer forms a tight group (illustrated as S1, S2, and S3 from the same manufacturer), which means good consistency among of batches. From the score plot, it can be seen that the structure characteristics of Z-52 and S1were obviously different from L647 and YT.

**Figure 6:**
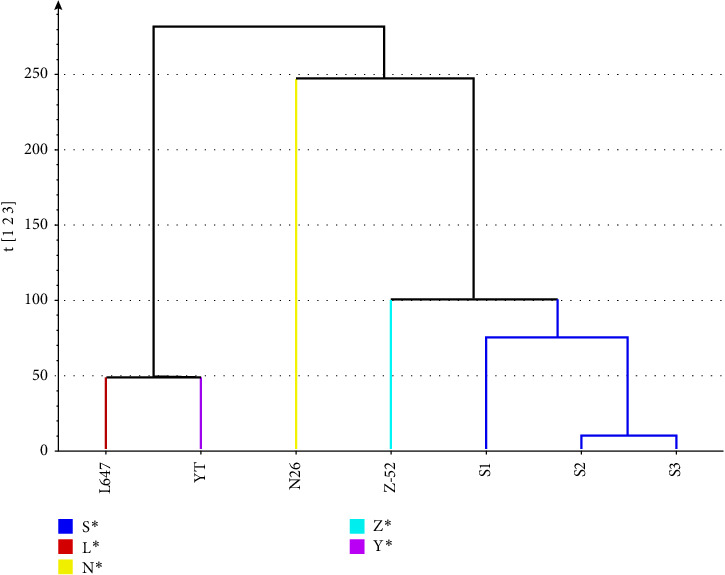
The dendrogram of all seven CP-52 samples.

The dendrogram illustrates the hierarchical cluster distance analysis of all seven CP-52 samples generated by the PCA of the ^1^H NMR spectra (see Figure 6). It showed that the CP-52 produced by different manufacturers form three groups. S1, S2, S3, and Z-52 were classified as one group; L647 and YT were classified as another group; and N26 was classified as the third group. The result was consistent with the above structure characterization results using LR-APCI-MS. It can be explained when the carbon chain length distribution shifted, and the structural elements in the CP-52 mixture will also be shifted, which would result corresponding shift in ^1^H NMR spectra.

**Figure 7:**
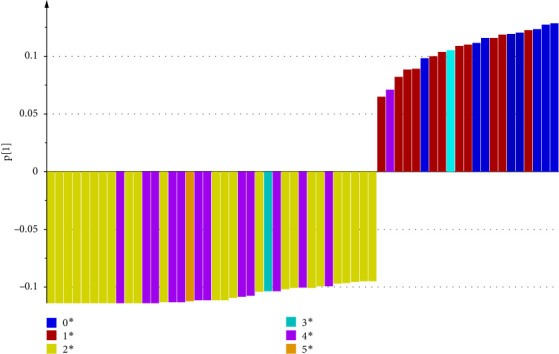
The resonances responsible for products discrimination in the loading plot.

The loading plot shows the structural elements responsible for such discrimination (see Figure 7). Resonances around *δ*0.73, *δ*0.83, and *δ*0.85 ranked as significant positive factor, and resonances around *δ*2.23, *δ*2.71, and *δ*2.74 ranked as significant negative factor, as Z-52 and S1 are in the positive zone of PC1 axis, while L647 and YT are in the negative zone of PC1 axis. So, it can be deduced that Z-52 and S1 contain more amount of R-CH_3_(from chemical shift at *δ*0.73, *δ*0.83, and *δ*0.85, the structural moieties can be more accurately attributed to R′-CH_2_-CH_3_, i.e., methyl groups with vicinal groups of CH_2_), while L647 and YT contain more amount of R_1_-CH_2_-R_2_ (from chemical shift at *δ*2.23, *δ*2.71, and *δ*2.74, the structural moieties can be more accurately attributed to R′-CCl_2_-CH_2_- CCl_2_-R′ and R′-CCl_2_-CH_2_-CCl_3_, i.e., methylene groups with vicinal groups of CCl_2_ or CCl_3_ in both sides).

Previous research indicated that the short-chain, heavily chlorinated paraffin appears to have more ecotoxicity and carcinogenicity than that of longer-chain, lightly chlorinated paraffin [6]. From this view, L647 and YT may have more toxic and carcinogenic than Z-52 and S1, and Z-52 and S1 were preferred technical products from ^1^H NMR result. However, considering that Z-52 contains 6.47% of SCCPs reflected by above LR-APCI-MS test, it is also excluded as the preferred technical CPs for environmental concerns. By comprehensively considering for LR-APCI-MS and ^1^H NMR result, S1 may be the preferred technical CPs for industrial use.

## 4. Conclusions

We demonstrated here a cost-effective method for structure characterization of technical CPs. By uisng direct injection low resolution mass spectrometry with data deconvolution, chain length distribution and homologous distribution of C_n_Cl_m_ in technical CPs mixture can be delineated, and by using ^1^H NMR with chemometrics tools, the chlorine distribution pattern can be elaborated. Combining the two analytical strategies, structural information at different levels that related to CPs' environmental toxicities were revealed. The method is expected to be easily applied in both industry and academia, aiming for quality control of technical CPs, to permit only CPs of nontoxic or noncarcinogenic to enter industrial use.

## Data Availability

All data generated or analyzed during this study are included within the article.

## Supporting Information

Supporting Information 1 Figure S1: The mass spectra of the seven CP-52 samples.

Supporting Information 2 Table S1: The arraryed mz value of [M+Cl]-in the simulatied all possible saturated congeners in the range of C12–C19.

Supporting Information 3 Table S2: The congener distribution and profile of each CP-52 deduced from peak fitting of mass spectrum data.
