# Supplementary material for: Structure Characterization and Products Control of Technical Chlorinated Paraffins by Direct Injection Mass Spectrometry With Data Deconvolution and 1H NMR With Chemometrics Tools
Source: J Anal Methods Chem. 2025 Apr 19;2025:1180345. doi: 10.1155/jamc/1180345 (PMC12033057; doi:10.1155/jamc/1180345)
Supplement: Supporting Information 1 — Figure S1: The mass spectra of the seven CP-52 samples. [file 1180345.f1.docx]

**Figure S1.** The mass spectra of the seven CP-52 samples


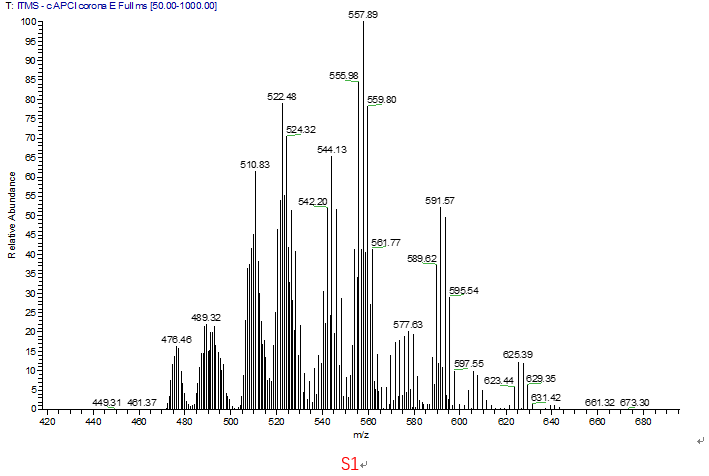


The mass spectra of CP-52 sample S1


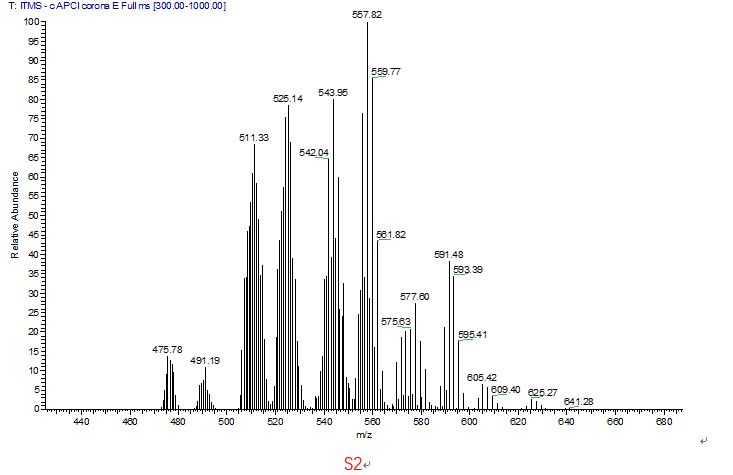
The mass spectra of CP-52 sample of S2


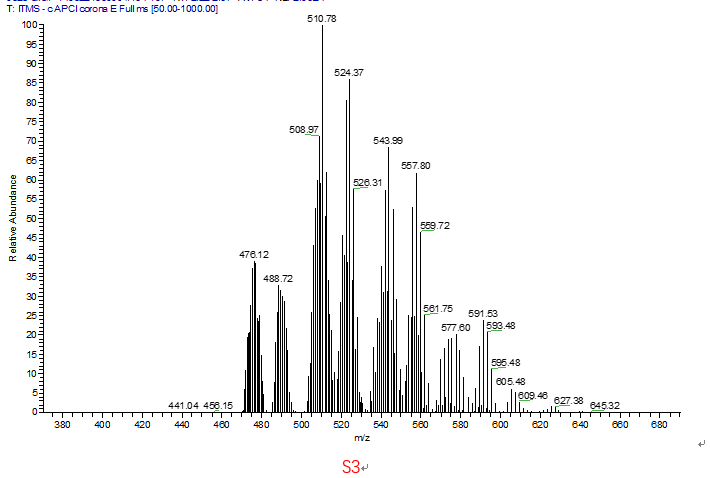
The mass spectra of CP-52 sample of S3


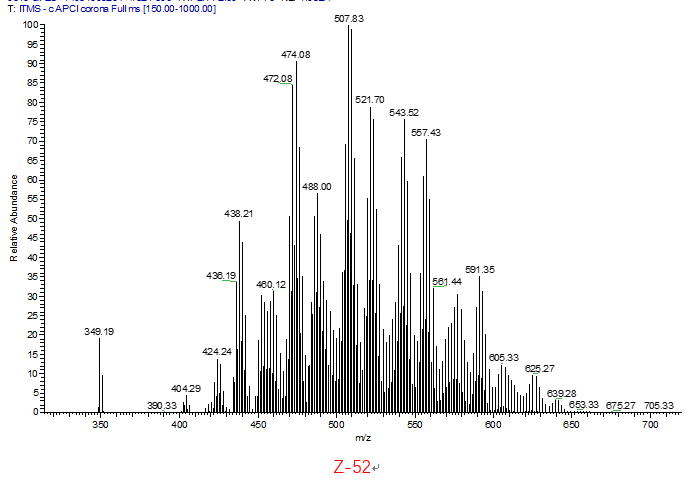
The mass spectra of CP-52 sample of Z-52


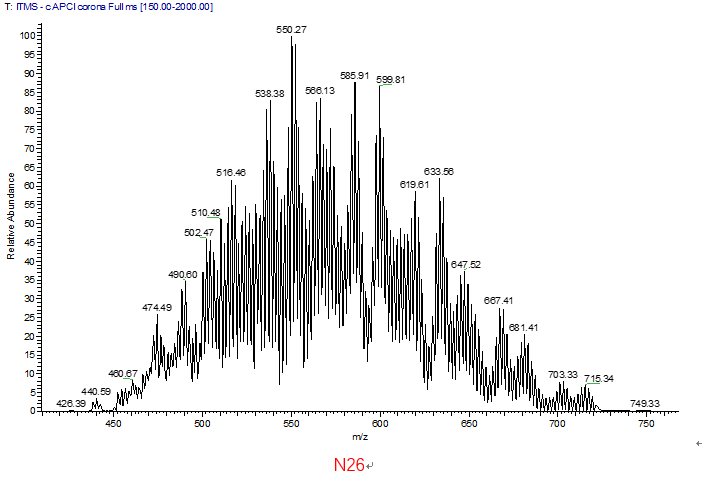
The mass spectra of CP-52 sample of N26


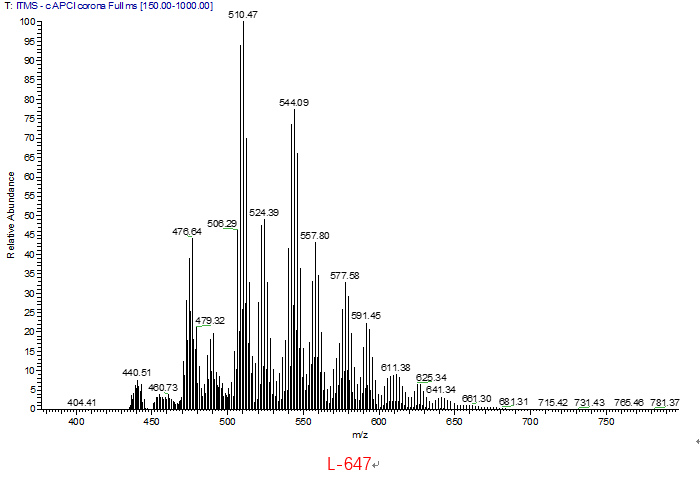
The mass spectra of CP-52 sample of L-647


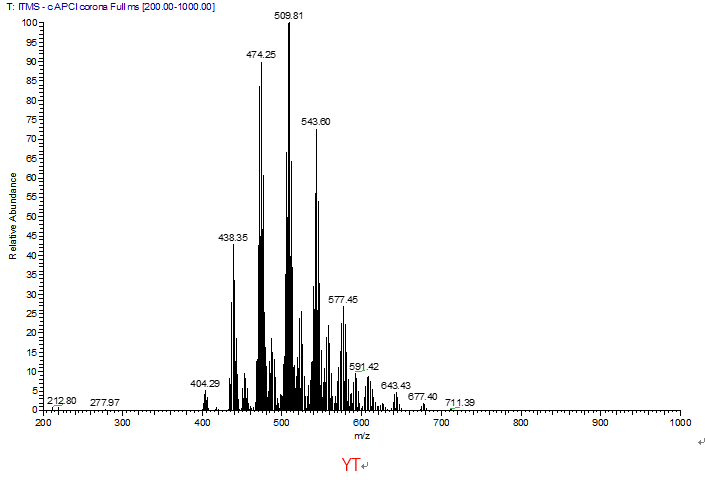


The mass spectra of CP-52 sample of YT
